# Integrated bioinformatics analyses identifying potential biomarkers for type 2 diabetes mellitus and breast cancer: In SIK1-ness and health

**DOI:** 10.1371/journal.pone.0289839

**Published:** 2023-08-09

**Authors:** Ilhaam Ayaz Durrani, Attya Bhatti, Peter John

**Affiliations:** Department of Healthcare Biotechnology, Atta ur Rehman School of Applied Biosciences (ASAB), National University of Sciences and Technology (NUST), H12, Islamabad, Islamabad Capital Territory, Pakistan; BMSCE: BMS College of Engineering, INDIA

## Abstract

The bidirectional causal relationship between type 2 diabetes mellitus (T2DM) and breast cancer (BC) has been established by numerous epidemiological studies. However, the underlying molecular mechanisms are not yet fully understood. Identification of hub genes implicated in T2DM-BC molecular crosstalk may help elucidate on the causative mechanisms. For this, expression series GSE29231 (T2DM-adipose tissue), GSE70905 (BC- breast adenocarcinoma biopsies) and GSE150586 (diabetes and BC breast biopsies) were extracted from Gene Expression Omnibus (GEO) database, and analyzed to obtain differentially expressed genes (DEGs). The overlapping DEGs were determined using FunRich. Gene Ontology (GO), Kyoto Encyclopedia of Genes and Genomes (KEGG) and Transcription Factor (TF) analyses were performed on EnrichR software and a protein-protein interaction (PPI) network was constructed using STRING software. The network was analyzed on Cytoscape to determine hub genes and Kaplan-Meier plots were obtained. A total of 94 overlapping DEGs were identified between T2DM and BC samples. These DEGs were mainly enriched for GO terms RNA polymerase II core promoter proximal region sequence and its DNA binding, and cAMP response element binding protein, and KEGG pathways including bladder cancer, thyroid cancer and *PI3K-AKT* signaling. Eight hub genes were identified: *interleukin 6 (IL6)*, *tumor protein 53 (TP53)*, *interleukin 8 (CXCL8)*, *MYC*, *matrix metalloproteinase 9 (MMP9)*, *beta-catenin 1 (CTNNB1)*, *nitric oxide synthase 3 (NOS3)* and *interleukin 1 beta (IL1β)*. *MMP9* and *MYC* associated unfavorably with overall survival (OS) in breast cancer patients, *IL6*, *TP53*, *IL1β and CTNNB1* associated favorably, whereas NOS3 did not show any correlation with OS. *Salt inducible kinase 1 (SIK1)* was identified as a significant key DEG for comorbid samples when compared with BC, also dysregulated in T2DM and BC samples (adjusted p <0.05). Furthermore, four of the significant hub genes identified, including *IL6*, *CXCL8*, *IL1B* and *MYC* were also differentially expressed for comorbid samples, however at p < 0.05. Our study identifies key genes including *SIK1*, for comorbid state and 8 hub genes that may be implicated in T2DM-BC crosstalk. However, limitations associated with the insilico nature of this study necessitates for subsequent validation in wet lab. Hence, further investigation is crucial to study the molecular mechanisms of action underlying these genes to fully explore their potential as diagnostic and prognostic biomarkers and therapeutic targets for T2DM-BC association.

## Introduction

Breast cancer signaling pathways are governed by two functionally distinct, antagonistic sets of genes: tumor suppressors and oncogenes. Genetic alterations in these master regulators result in the ‘switching off’ of tumor suppressor genes such as *tumor protein (p53)*, *breast cancer genes 1 and 2 (BRCA 1/2)*, *phosphatase and tensin homolog deleted in chromosome 10 (PTEN)* and *retinoblastoma gene (RB1)*, and ‘turning on’ of proto-oncogenes into oncogenes like transcription factor *MYC*, *phosphatidylinositol-4*,*5-Bisphosphate 3-Kinase catalytic subunit A (PIK3CA)* and *receptor tyrosine protein kinase ErbB2*, mediating a series of molecular and physiological changes leading to carcinogenesis in the mammary gland [1, 2]. Interestingly, several of these genes have also been reportedly dysregulated in diabetes, particularly type 2 diabetes mellitus (T2DM), potentially laying down the foundation for the molecular bases underlying the two-way relationship between these two complex and multifactorial diseases. This in turn supports the growing body of epidemiological evidence reporting the incidence of T2DM in breast cancer patients and also cases of T2DM induced breast cancer [3].

Breast cancer (BC) is the most frequently reported cancer amongst females [4], worldwide, and diabetes too is emerging globally as a pandemic [5, 6]. Hence both these diseases are leading causes of national and international health concern. At this rate, reports of increased breast cancer incidence in diabetic patients and the worsening of prognosis in breast cancer patients with T2DM onset, adds to the health burden, and requires immediate attention of scientists worldwide [7, 8].

Hence, this study aimed to investigate the molecular mechanisms underlying the type 2 diabetes mellitus and breast cancer association by utilizing bioinformatics methods to identify key molecular players commonly dysregulated in both T2DM and breast cancer and potentiate biomarker discovery based on identifying differential and common gene expression patterns between samples derived from T2DM affected adipose tissue, breast adenocarcinoma biopsies and comorbid patients.

## Materials and methods

This paper adopted an ‘integrated *in silico* analyses’ approach for the identification of potential biomarkers common to T2DM and breast cancer.

### Selection of microarray and RNA-seq. data

The T2DM series GSE29231 is based on the GPL6947 platform Illumina HumanHT-12 V3.0 expression beadchip, BC series GSE70905 on GPL4133 platform Agilent-014850 Whole Human Genome Microarray 4x44K G4112F (Feature Number version) [9, 10] and GSE150586 series on GPL24676 Illumina NovaSeq 6000 (Homo sapiens). Table 1 describes further details for each of these series. To the best of our knowledge, any combination of these expression series has not been previously analyzed for T2DM-BC cross disease comparison. All the expression data analyzed here is freely accessible on the GEO database website.

**Table 1:** GEO series utilized for this study. The table details the disease sample size and microarray/RNA-seq. platform. T2DM: type 2 diabetes mellitus; BC: breast cancer; D: diabetes.

#	Series ID	Description	Platform	Disease	Ref.
1	Series GSE29231	Samples of visceral adipose tissue taken from 3 female T2DM patients and 3 female healthy controls (3 biological x 4 technical replicates)	GPL6947 platform Illumina HumanHT-12 V3.0 expression beadchip	T2DM	[9]
2	Series GSE70905	47 Breast adenocarcinoma and 47 adjacent paired normal samples	GPL4133 platform Agilent-014850 Whole Human Genome Microarray 4x44K G4112F (Feature Number version)	BC	[10]
3	Series GSE150586	6 samples from comorbid patients (diabetes with breast cancer) and 6 breast cancer samples	GPL24676 platform Illumina NovaSeq 6000 (*Homo sapiens*)	D+BC	[11]

### Differential gene expression analysis

Each of the series were analyzed using the GEO2R tool on NCBI GEO website to obtain differentially expressed genes (DEGs) between diseased and normal/control samples (https://www.ncbi.nlm.nih.gov/geo/geo2r/). An (adjusted) *p* value of < 0.05 and an absolute value of log of fold change of 1, or more (|log FC| ≥ 1) were utilized as selection criteria for identifying significant DEGs. The Venn diagram analysis was performed to determine an overlap between DEGs from both series using the FunRich software (http://www.funrich.org/), which is a user friendly tool that allows for graphical representation of the data [12].

### DEG GO and KEGG analyses

The Gene Ontology (GO) and KEGG analyses were performed using EnrichR software (https://maayanlab.cloud/Enrichr/). EnrichR is an online enrichment analysis tool that analyzes gene sets for various aspects of biological knowledge [13]. Functional enrichment of the overlapping significant DEGs were obtained for three GO terms classified as biological process (BP), molecular function (MF), and cellular component (CC). Kyoto Encyclopedia of Genes and Genomes (KEGG) pathway enrichment was also determined. KEGG associates a set of genes with biological functions such as signaling, pathways and interacting networks of proteins [14].

### PPI network construction

The protein-protein interaction (PPI) network for the cross disease analysis -common DEGs were constructed using Search Tool for the Retrieval of Interacting Genes (STRING) software (https://string-db.org/), by introducing the DEGs list onto the software interface online. PPI pairs with a combined score of 0.4 or above were extracted to generate the networks.

### Hub gene identification

The PPI network topology was then visualized using Cytoscape software (https://cytoscape.org/), and the average node degree parameter was calculated using the ‘Network Analyzer’ feature. Genes corresponding to a node degree of 10 and above were considered as hub genes.

### Survival analysis of hub genes

Kaplan Meir plot was generated using the Kaplan-Meir plotter (http://kmplot.com/analysis/) to gain insight into the prognostic value of each hub/key gene in terms of breast cancer survival outcome. Kaplan-Meir plotter is an online tool that features the option to assess the prognostic relevance of about 54000 genes in various types of cancers, including expression data for 4929 breast cancer patients [15, 16].

**Fig 1:**
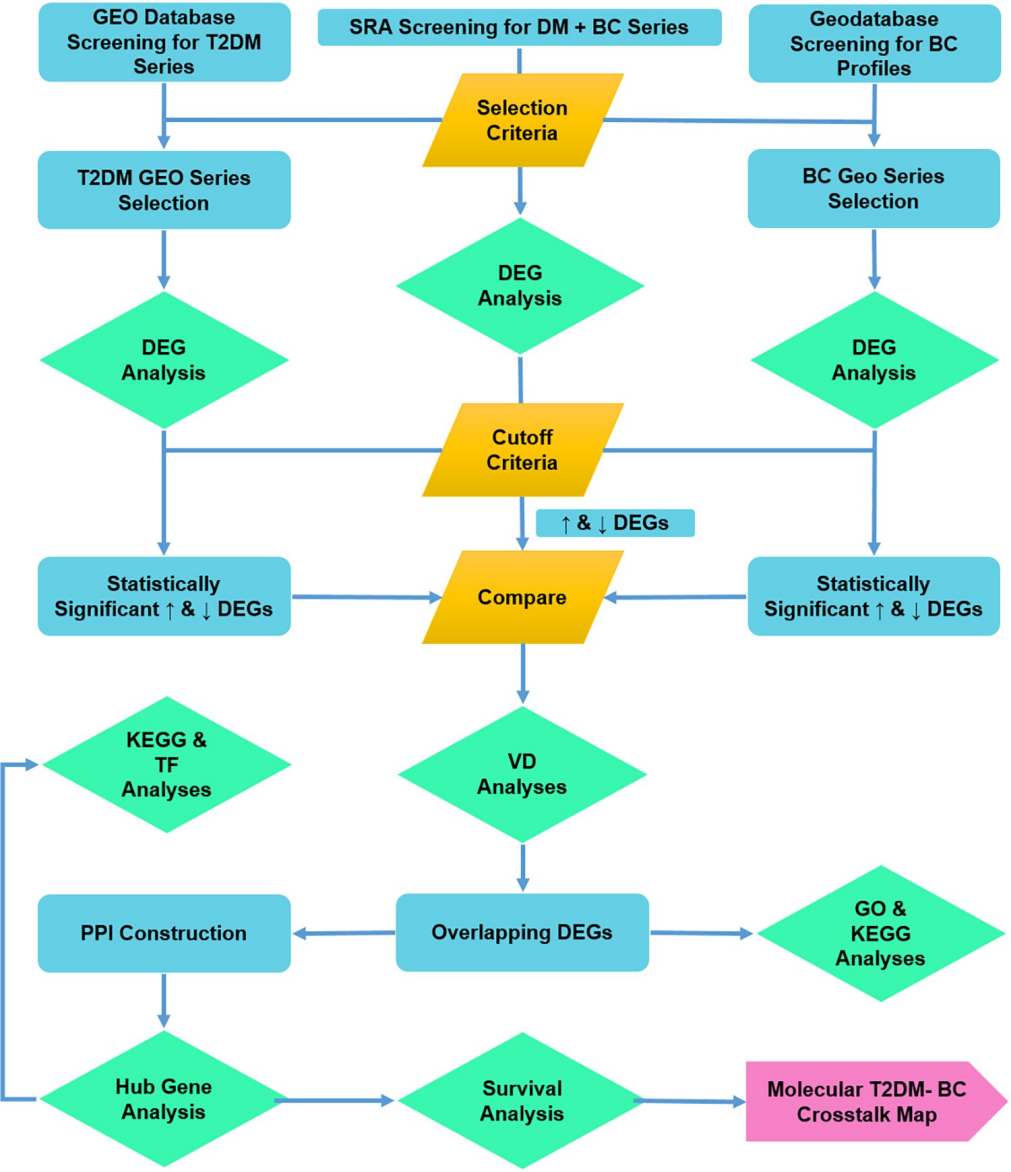
Flow diagrammatic representation of methodology. The boxes in green represent analyses, yellow-conditions applied and blue for immediate outcome/ subsequent process step. The pink box indicates the final outcome.

The methodology is summarized in Fig 1.

## Results

### Identification of DEGs

Two series containing microarray data, were selected, one each for T2DM and breast cancer, for cross disease analysis. GSE29231 consisted of visceral adipose tissue of 3 female T2DM patients and 3 healthy controls and GSE70905 with 47 BC and 47 paired healthy control samples as summarized in Table 1.

DEG analysis for each of these series was carried out by comparing the disease samples with corresponding normal samples and the list of differentially expressed genes was extracted according to the criteria of adjusted *p* < 0.05 and |log FC| ≥ 1, as shown in Table 2. A total of 1187 significant DEGs were screened for T2DM adipose tissue samples, which consisted of 857 up-regulated and 330 down-regulated DEGs. For the breast adenocarcinoma samples, a total of 3003 significant DEGs were obtained, of which 1290 were up-regulated and 1713 were down-regulated. As part of subsequent validation, DEG analysis of GSE150586 presented with a total of 60 statistically significant DEGs, of which 33 were found to be up-regulated and 27 down-regulated. The top ten up- regulated and down-regulated DEGs for each of the analyses are also listed (Table 2).

**Table 2:** Differential gene expression analysis. The results show the number of differentially expressed genes (DEGs) for each series analyzed, along with the top 10 most significant up-regulated and down-regulated DEGs.

#	Series ID	Total Potential DEGs	Total Significant DEGs	Up-Regulated Genes	Down-Regulated Genes	Top 10 Up-Regulated Genes	Top 10 Down-Regulated Genes
1	GSE29231	48803	1187	857	330	*LGALS8; S100A12; KDM5D; MIOX; ISLR; VNN2; CAMK2B; GP9; NEUROD2; FOS*	*FRG2B; CD207; AMPD2; ZNF8; SLC24A5; SLC5A12; FBXO33; KRTAP23-1; ABCB4; A1CF*
2	GSE70905	45015	3003	1290	1713	*SPATS2L; PLXNA4; SPDYE3; SP1; DOK5; TAX1BP3; ARHGAP26; ZSCAN29; ADAMTS10; TERT;*	*SHCBP1 SAA4; LINC00152; MIGA1; EYA4; CHADL; CDK15; EXOC7; CCL23; PYROXD1*
	GSE150586	19231	60	33	27	*UGT2B28; ALOX15B; SLC30A8; LBP; FIBCD1 CEACAM5; CLCA2; SPINK8; SERHL2; CRYM*	*SERPINA11 CYP2B7P; SYT13; CYP4F30P; PHGR1; FOSB; SOCS3; NR4A3; DLX2; LRP2*

### Venn diagram analysis

**Fig 2:**
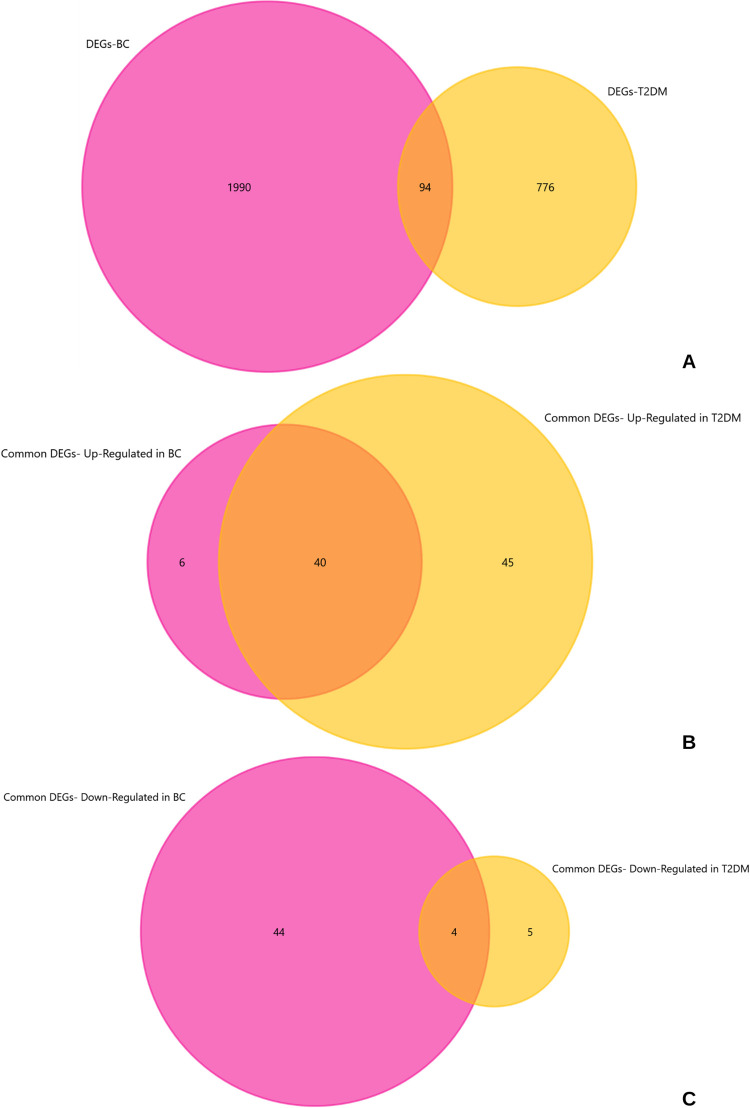
Venn diagram analysis for T2DM -adipose tissue and breast adenocarcinoma (BC) samples. The yellow circle represents DEGs obtained from DEG analysis on T2DM samples and the pink circle represents DEGs from BC samples, similarly. **A**- The intersection represents DEGs common to T2DM and BC. **B**- The intersection represents up-regulated DEGs common to both diseases. **C**- The intersection represents down-regulated DEGs common to both diseases.

The overlapping DEGs between series were determined with Venn diagram analysis, where the intersection represented DEGs common to both the series compared, in each analysis performed. For T2DM, 1187 DEGs and for BC, 3003 DEGs were imported into FunRich software, which were subsequently mapped to 835 (/870) and 1915 (/2084) genes respectively, removing any repetitions in DEGs. There were a total of 94 DEGs common to both T2DM adipose tissue and breast adenocarcinoma samples as shown in Fig 2A, of which 40 DEGs were commonly up-regulated (Fig 2B) and 4 commonly down-regulated in both diseases (Fig 2C).

These common DEGs are listed in Table 3.

**Table 3:** Venn diagram analysis. The 94 DEGs common to both T2DM and BC are listed. The expression of each of the DEGs in both diseases is also indicated. DEGs: differentially expressed genes; T2DM: type 2 diabetes mellitus; BC: breast cancer.

T2DM- BC Series Compared	No. of Common DEGs	Expression Pattern in T2DM		Expression Pattern in BC	
		↑	↓	↑	↓
T2DM- BC	94	*KDM5D; SOD3; MIOX; POMZP3; TAPBP; MCL1; CRTC1; MMP9; CXCL8; MAP2K3; SERPINB2; MYC; MSR1; S100A8; CEL; TPM4; ELN; PTMS; SLC11A1; RBMS1; TPM3; ZC3HAV1; TNC; WDR1; FGFR1; MAPK8IP3; RP2; CALD1; AGRN; GEM; ARID4B; MRGPRF; TYMP; SIK1; FAM118A; ACTRT1; HIPK2; SCAMP1; FSTL3; ITGAX; CCDC86; ADAMTS1; SP2; SYN2; PRMT2; SNCA; PMP22; RFX1; CDKN1A; NOS3; NUP98; EPOR; LDOC1L; DYNLT3; TP53; GGCX; WTAP; SF3A2; GRASP; SPATS2L; PTPRE; CYSLTR1; EPN1; BCORL1; PLPP1; WDR4; TUBA1C; HSPA1A; RBPJ; FAM222B; CBX4; FKBP1A; FGR; CTNNB1; IL1B; WRNIP1; MYL9; TSPAN13*, *JUNB*, *STX11; BCL3; SLC25A20; IL6; EXOC4; GTPBP8*	*CDC14A; SMAD5; KRTAP3-2; RAX; PPP2R1B; PER3; MCOLN3; RPL21; CHURC1*	*KDM5D; SOD3; JUNB; TAPBP; CEL; CRTC1; MMP9; IL1B; MYL9; MYC; FGR; ZC3HAV1; WDR1; TPM3; CALD1; CBX4; GEM; ARID4B; SIK1; CDKN1A; NUP98; TP53; SP2; SYN2; SPATS2L; GGCX; CYSLTR1; BCORL1; WDR41; HSPA1A; EPN1; FKBP1A; WRNIP1; EXOC4; TSPAN13; RBPJ; SLC25A20; RPL21; SMAD5; ITGAX; HIPK2; PPP2R1B; MCOLN3; TNC; CHURC1; SCAMP1*	*MIOX; POMZP3; MCL1; BCL3; IL6; CXCL8; MAP2K3; SERPINB2; MSR1; S100A8; TPM4; ELN; PTMS; SLC11A1; RBMS1; FGFR1; PRMT2; MAPK8IP3; PER3; AGRN; MRGPRF; TYMP; RP2; ACTRT1; FSTL3; CCDC86; PMP22; FAM118A; SNCA; ADAMTS1; RFX1; NOS3; EPOR; FAM222B; LDOC1L; DYNLT3; WTAP; SF3A2; GRASP; PTPRE; PLPP1; TUBA1C; CTNNB1; STX11; GTPBP8; CDC14A; KRTAP3-2; RAX*

### Functional enrichment analyses

**Fig 3:**
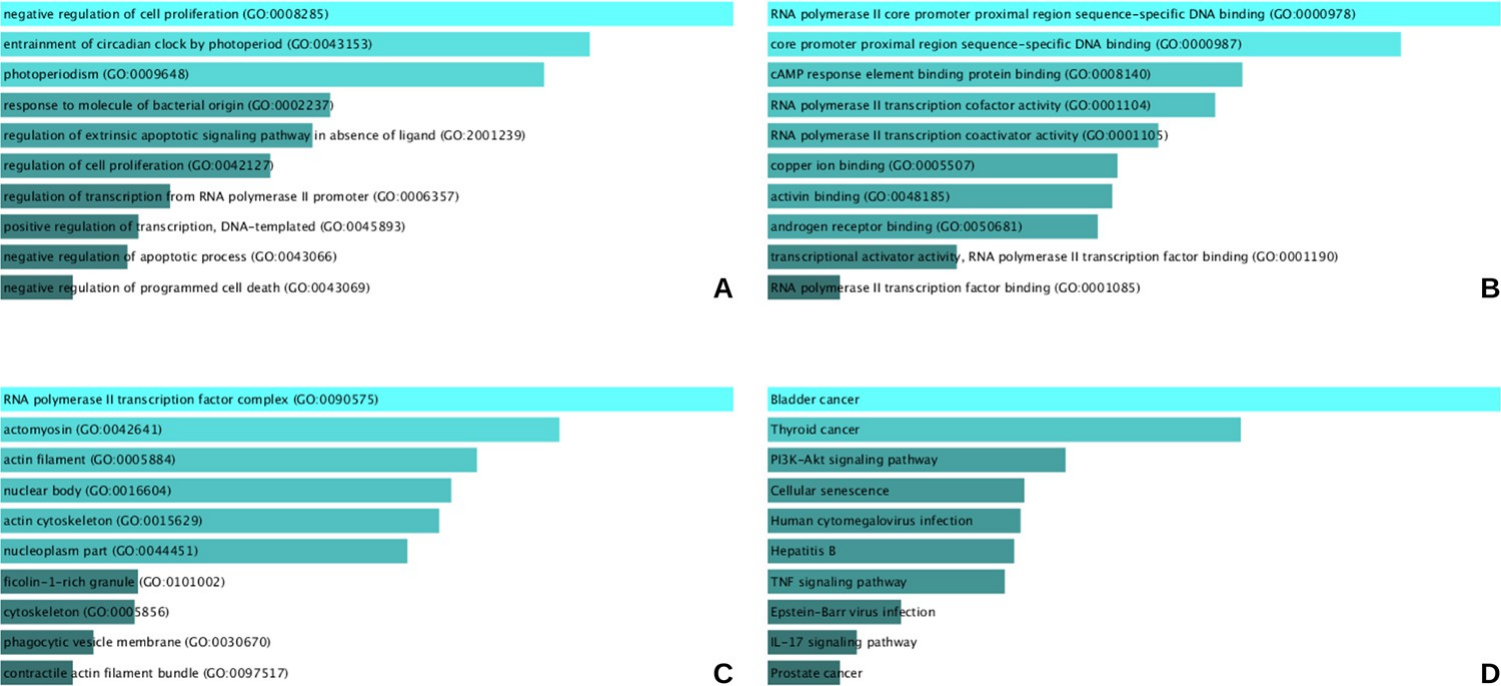
Functional and pathway enrichment analyses. The most significant results are presented from top to bottom. **A**- Shows the results for GO term biological processes, **B**- GO term molecular function, and **C**- GO term cellular component. **D**- Most significantly results for KEGG pathways.

Functional and pathway enrichment analyses showed that the common DEGs between T2DM and BC series were enriched for GO category ‘biological processes’ such as negative regulation of cell proliferation, entrainment of the circadian clock by photoperiod and photoperiodism, ‘molecular functions’ including RNA polymerase II core promoter proximal region sequence- specific DNA binding, core promoter proximal region sequence and cAMP response element binding protein, and ‘cellular components’ RNA polymerase II transcription complex, actin myosin and actin filament. The top three significantly enriched KEGG pathways were bladder cancer, thyroid cancer and *PI3K-AKT* signaling. Additionally, cellular senescence, *TNF*- signaling and *IL17* signaling pathways were also amongst the significantly enriched results, as shown in Fig 3A–3D.

### PPI network construction

**Fig 4:**
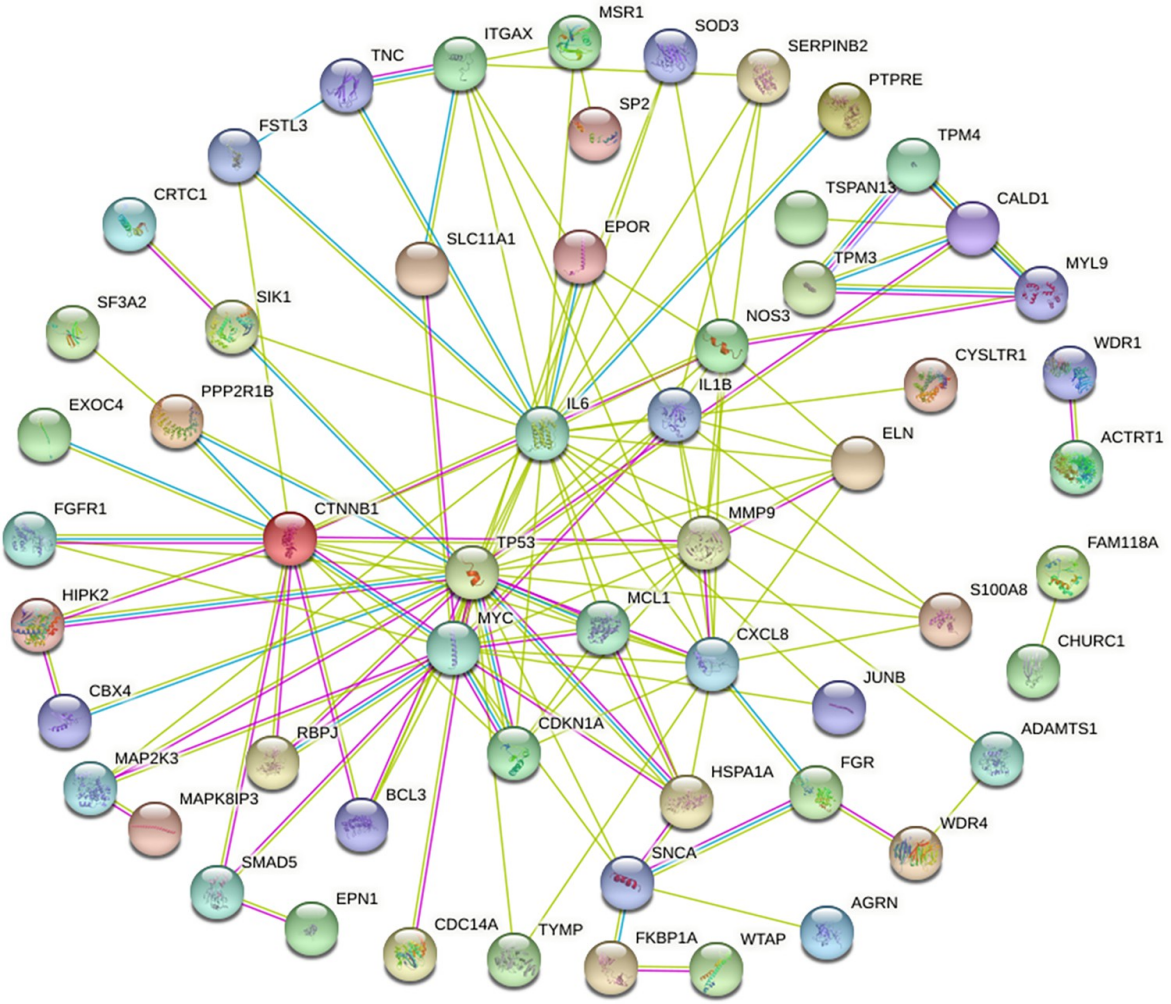
Protein- protein interaction network for common DEGs. All disconnected nodes were removed from the network. The sphere represents node (protein encoded by the gene) and the line depicts interaction. The color of the line is representative of the source of evidence for interaction, including text mining (light green), gene neighborhood (dark green), experimentally determined (magenta), and curated databases (blue). The structure within sphere represents the availability of 3D structure of the protein.

STRING software was utilized to predict interactions between the common DEGs obtained from comparing the singular disease’s DEGs lists. A total of 94 nodes were imported into the online software to generate a PPI network with 146 edges at a confidence of 0.4 and average node degree of 3.11, as shown in Fig 4.

### Hub gene identification

**Fig 5:**
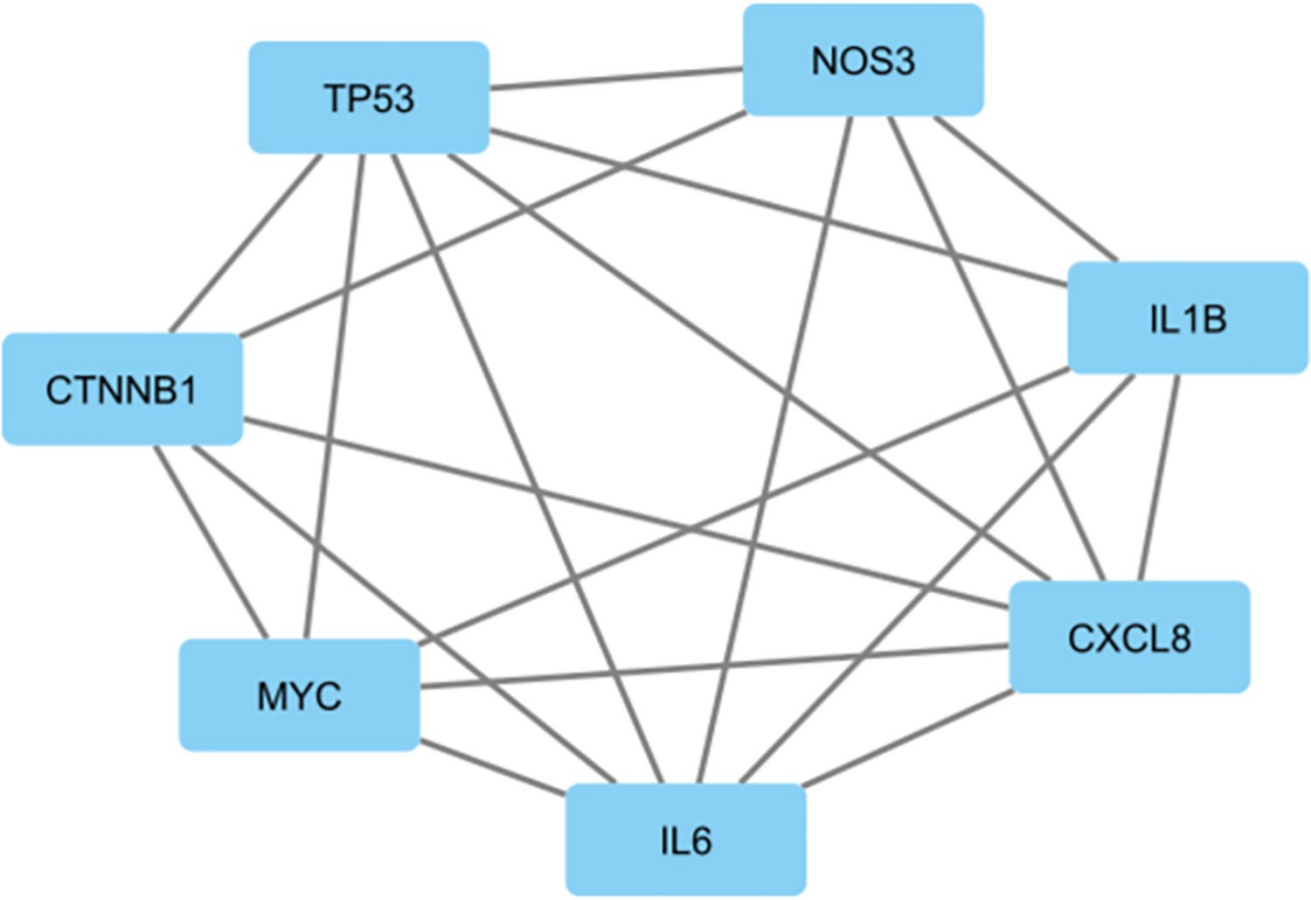
Sub-network of hub genes. The blue rectangle represents the gene and the grey line depicts interaction between a pair of hub genes.

The PPI network was imported into Cytoscape software and the nodes were evaluated for their node degrees. Nodes with a degree ≥ 10 were identified as hub genes. These included *tumor protein 53 (TP53)* and *interleukin (IL6)* with the highest node degrees of 25 each, followed by *interleukin 8 (CXCL8)* and *MYC*, both at 18, *matrix metallopeptidase 9 (MMP9)* and *beta- catenin 1 (CTNNB1)* at 15, and *nitric oxide synthase 3 (NOS3)* and *interleukin 1 beta (ILβ1)* at a degree of 10 each. A sub-network of these hub genes is represented in Fig 5.

### Hub gene survival analysis

**Fig 6:**
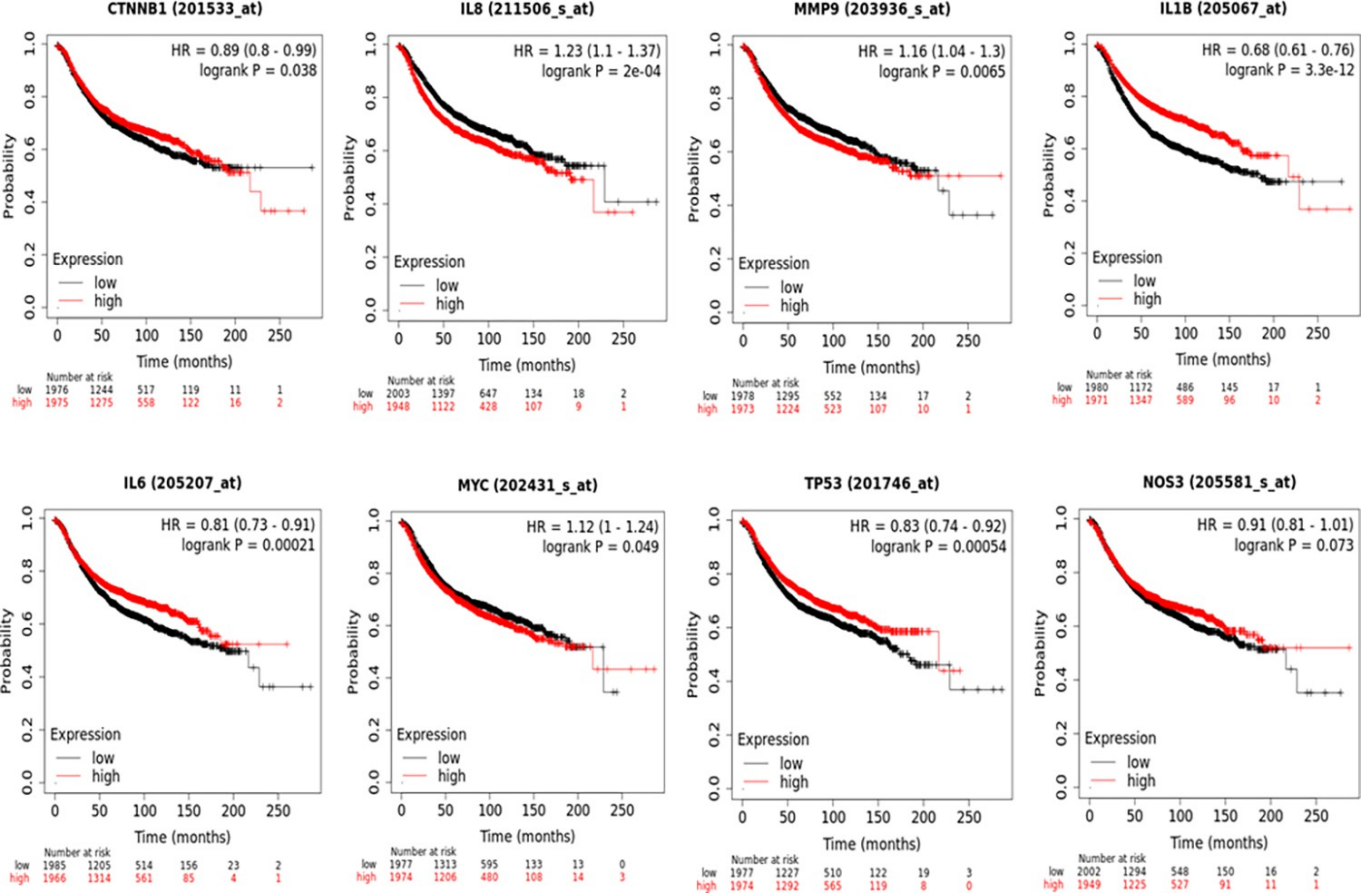
Kaplan-Meier plots. These high vs. low gene expression curves represent overall survival in breast cancer patients for each of the hub genes identified.

To study the prognostic value of these hub genes in breast cancer prognosis, survival analyses were performed using breast cancer patients’ data at the Kaplan- Meier plotter platform. The curves obtained as shown in Fig 6 indicate that *MMP9*, *MYC* and *IL8* associate unfavorably with overall survival (OS) in breast cancer patients, whereas, *IL6*, *TP53*, *IL1β* and *CTNNB1* genes associate favorably. Additionally, *NOS3* does not show any correlation with overall survival.

Of these 8 genes, *TP53*, *MYC* and *IL1β* were upregulated in both T2DM and BC samples, whereas *MMP9*, *CTNNB1*, *IL6*, *CXCL8* and *NOS3* were all up-regulated in T2DM, but found to be down-regulated in BC samples.

### Pathway enrichment reanalysis for hub genes

**Fig 7:**
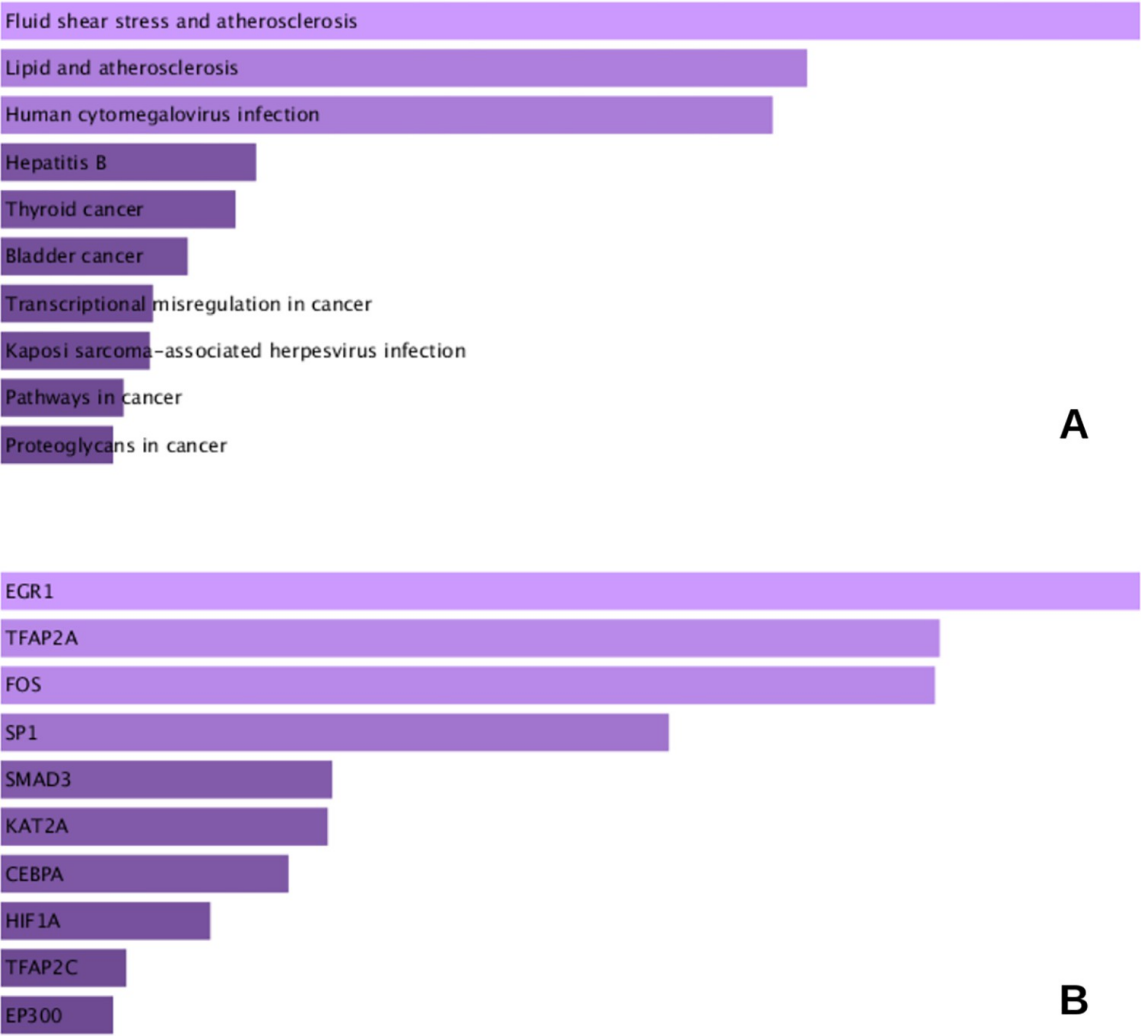
Functional enrichment re-analysis of hub genes. **A**- KEGG pathway enrichment **B**- Transcription factor enrichment analysis. The most significant results are presented from top to bottom.

The 8 hub genes were re-imported into the EnrichR tool, and the KEGG pathway analysis was performed along with enrichment analysis for transcription factors. Results are shown in Fig 7A and 7B.

Fig 7A shows that these hub genes were enriched for KEGG pathways such as fluid shear stress and lipid’s roles in atherosclerosis, human cytomegalovirus infection, hepatitis B and cancers such as thyroid, bladder and particularly breast cancer (not shown in the top 10 most significant results- S1 Table), along with other terms such as transcriptional mis-regulation in cancer and pathways in cancer. These hub genes were also enriched for transcription factors including *EGFR*, *TFAP2A*, *FOS*, *SP1*, *SMAD3*, *KAT2A*, *CEBPA*, *HIF1A*, *TFAP2C and EP300*, as depicted in Fig 7B.

### Validation analysis for comorbidity

**Fig 8:**
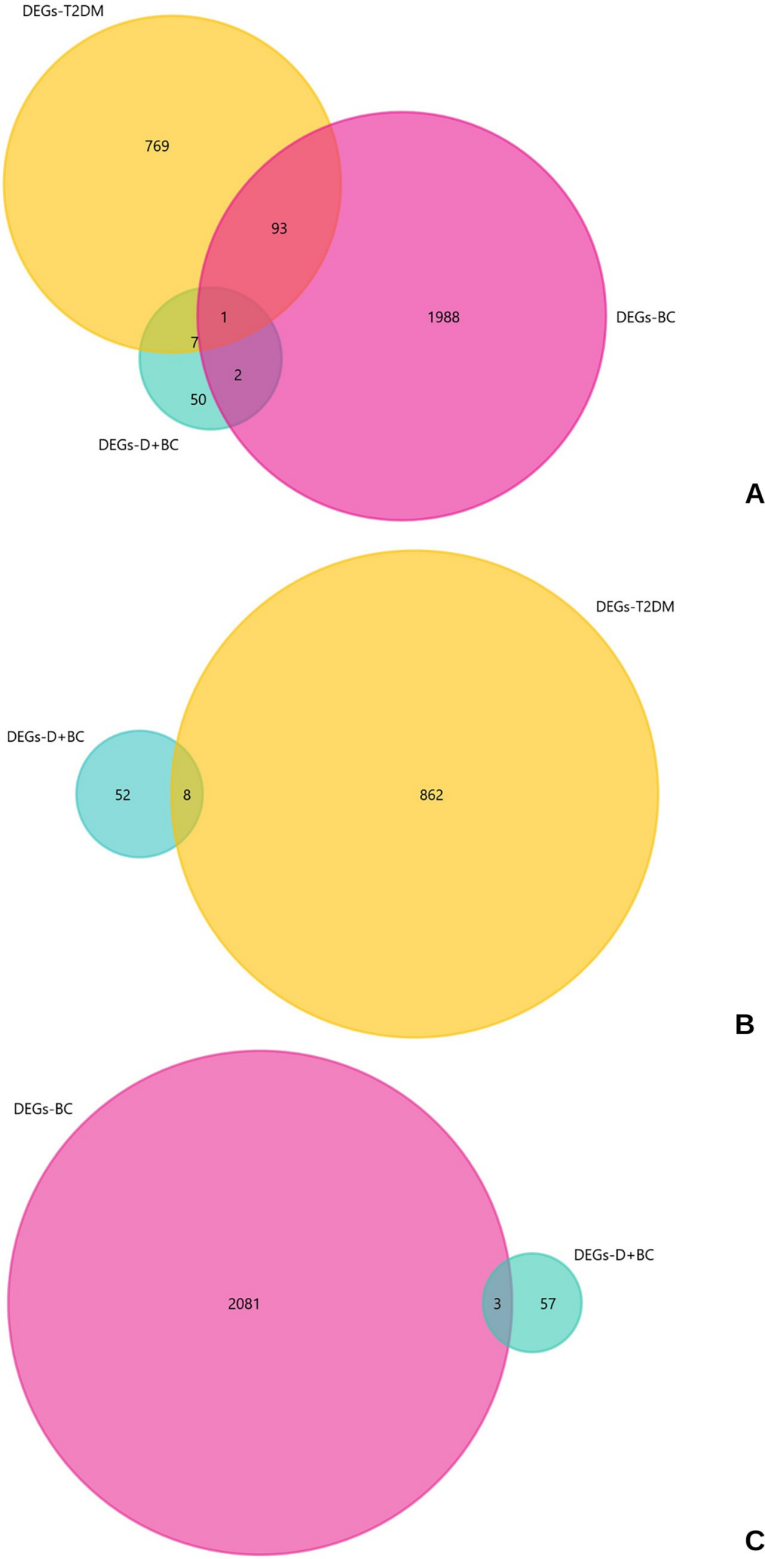
Venn diagram analysis for diabetes and breast cancer (D+BC) comorbid samples with T2DM -adipose tissue and breast adenocarcinoma (BC) samples. The yellow circle represents DEGs obtained from DEG analysis on T2DM samples, pink circle represents DEGs from BC samples, and sea green circle depict DEGs for D+BC samples, similarly. **A**- The intersection represents DEGs common to D+BC, T2DM and BC series. **B**- The intersection represents DEGs common to D+BC and T2DM diseased conditions. **C**- The intersection represents DEGs common D+BC and BC diseased states.

Sequentially, 60 DEGs from the DEG analysis of diabetes- breast cancer (D+BC) samples compared to BC samples, were imported into the FunRich software, and mapped to 48 (/60) genes. Venn diagram analysis showed only 1 DEG to be commonly differentially expressed between all three series- (T2DM, BC and comorbid D+BC, Fig 8A), whereas 8 DEGs overlapped between T2DM and comorbid state (Fig 8B), and 3 between BC and comorbid D+BC series (Fig 8C).

These overlapping DEGs are listed in Table 4.

**Table 4:** Venn diagram analysis. The DEGs common to the three diseased states in three combinations are listed. The expression of each of the DEGs in diseased state is also indicated. DEGs: differentially expressed genes; T2DM: type 2 diabetes mellitus, BC: breast adenocarcinoma, D+BC: diabetes and breast cancer comorbid state.

Series Compared	No. of Common DEG(s)	Expression Pattern in T2DM		Expression Pattern in BC		Expression Pattern in D+BC	
		↑	↓	↑	↓	↑	↓
T2DM- BC-D+BC	1	*SIK1*		*SIK1*			*SIK1*
T2DM- D+BC	8	*FOSB* *IER2* *JUN* *ATF3* *ADCY1* *SIK1* *JUND* *CSRNP1*					*FOSB* *IER2* *JUN* *ATF3* *ADCY1* *SIK1* *JUND* *CSRNP1*
BC- D+BC	3			*SIK1* *ERBB2*	*LBP*	*ERBB2* *LBP*	*SIK1*

**Fig 9:**
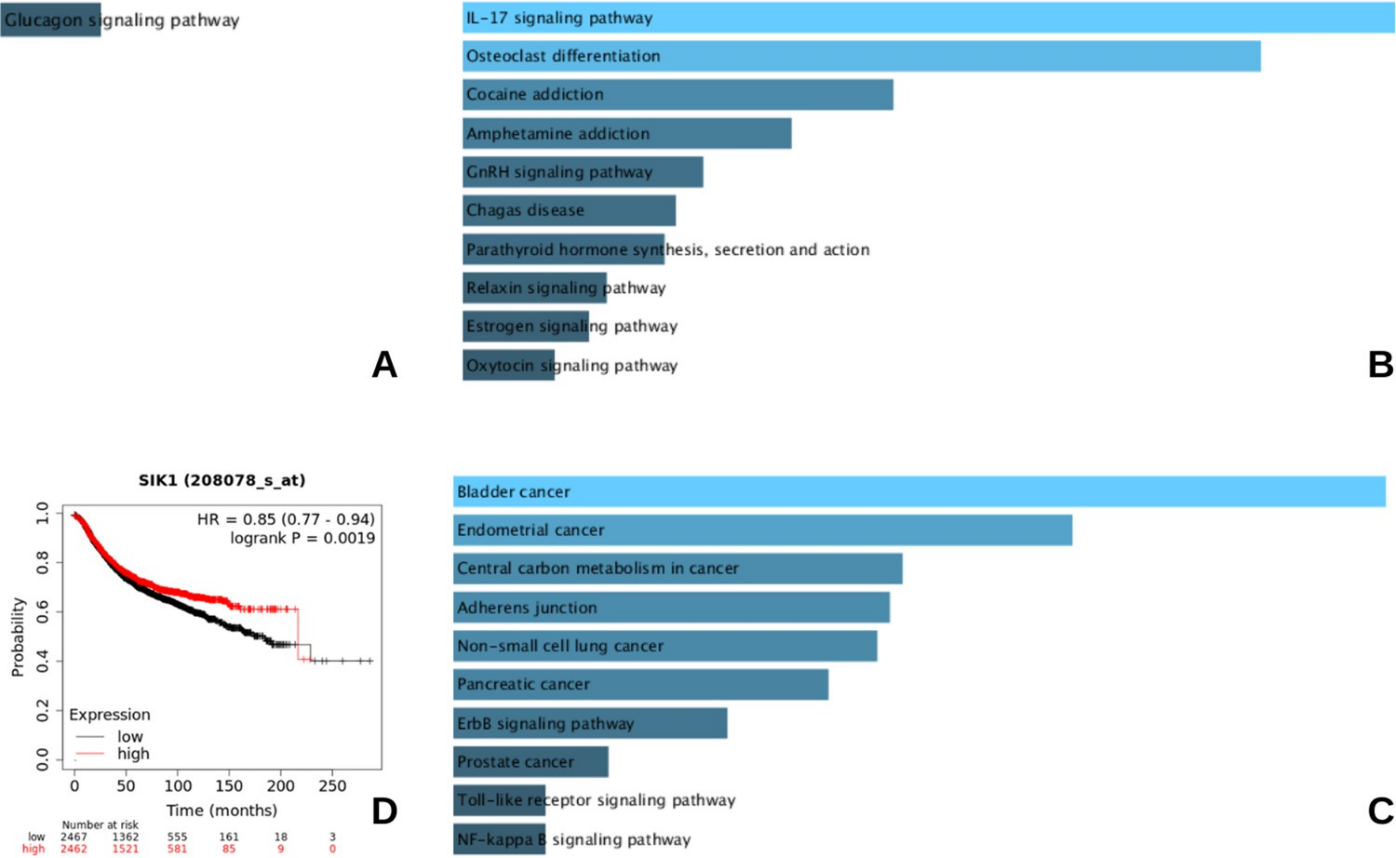
Pathway enrichment and survival analyses. **A**-**C** The most significant KEGG pathway results are presented from top to bottom for **A**- All three series, **B**- D+BC and T2DM series **C**- D+BC and BC series. **D**- Kaplan-Meier Plot for SIK1 gene. These high vs. low gene expression curves represent overall survival in breast cancer patients for the key gene identified.

Functional enrichment analysis for each of these sets of common DEGs were performed for category KEGG pathway. Results showed the singular DEG, *SIK1*, common to all three diseased states (T2DM, BC and comorbid D+BC) to be significantly implicated in glucagon signaling pathway (Fig 9A). The overlapping DEGs for T2DM-comorbid series were significantly enriched for KEGG terms *IL-17* signaling pathway, osteoclast differentiation and estrogen signaling, amongst others (Fig 9B), whereas the BC-comorbid overlapping DEGs were represented by KEGG pathways including several types of cancer, central carbon metabolism in cancer and *ErbB* signaling (Fig 9C). The prognostic relevance of *SIK1*, common to all three diseased states was also found out, as described previously for the hub genes (Fig 9D). The gene was shown to associate favorably with OS in BC patients.

## Discussion

This study aimed at identifying potential molecular markers dysregulated in both T2DM adipose tissue and in breast adenocarcinoma, irrespective of hormone receptor expression status. The difference in the number of DEGs obtained from the DEG analyses and the number of DEGs mapped onto FunRich software can be accounted for by the multiple splice variants for a single gene [17], detected during microarray expression analysis, but mapped to the same gene in FunRich. Results showed that there were a total of 94 DEGs common between both the diseases comparing microarray data from a T2DM adipose tissue derived series containing diseased and healthy control samples and another series composing breast adenocarcinoma (BC) samples as well healthy adjacent control tissues, Fig 2. These common DEGs consisted of 85 up-regulated and 9 down-regulated genes in T2DM, and 46 up-regulated and 48 down-regulated genes in breast cancer. These DEGs were consequentially analyzed for functional enrichment categories such as gene ontology, sub-divided into biological processes (BP), molecular function (MF) and cellular components (CC), and KEGG pathways. The results indicated these DEGs were enriched in GO BP terms such as cell proliferation and its negative regulation, photoperiod’s role in the entrainment of circadian clock and photoperiodism, response to molecule of bacterial origin, extrinsic apoptotic signaling regulation in the absence of ligand, transcription from RNA polymerase II promoter’s regulation, DNA-templated transcription’s positive regulation, and negative regulation of programmed cell death, particularly of apoptosis, as the most significant terms, as shown in Fig 3. These DEGs were involved in KEGG pathways implicated in bladder, thyroid and prostate cancers, *PI3K-AKT* signaling pathway, cellular senescence, human cytomegalovirus infection, Hepatitis B, *TNF* signaling pathway, Epstein-Barr virus infection, and *IL-17* signaling pathway.

The proteins corresponding to these DEGs were also utilized to construct a PPI network to study protein-protein interactions and identify the most connected proteins, central to the network, Fig 4. Eight hub genes were identified, including *TP53*, *MYC*, *IL1β*, *IL6*, *IL8*, *CTNNB1*, *MMP9* and *NOS3* (Fig 5). All of these genes were found to be up-regulated in T2DM adipose tissue samples, but were differentially expressed in BC samples. *TP53*, *MYC* and *IL1β* were up-regulated, whilst *IL6*, *IL8*, *CTNNB1*, *MMP9* and *NOS3* were under expressed as compared to healthy adjacent tissue.

To assess the prognostic significance of each of these hub genes, Kaplan-Meier plots were generated and showed that the overexpression of *TP53*, *IL1β*, *IL6* and *CTNNB1* associated favorably with OS (Fig 6). So, while the up-regulation of *TP53* and *IL1β* indicate favorable prognosis for these BC patients, the down-regulation of *IL6* and *CTNNB1* reflected the opposite. Additionally, *IL8*, *MMP9* and *MYC* associated unfavorably with OS, and hence, the overexpression of *MYC* may correspond to a poorer outcome, whereas the down-regulation of *IL8* and *MMP9* may translate into a better prognosis. *NOS3*, was shown to not be associated with prognosis.

Disease pathogenesis is the outcome of a multifactorial, complex series of metabolic and other molecular changes, and hence its prognosis prediction is inevitably a challenge. At the crux of this, it is important to understand that the expression of a single gene may not be sufficient to determine the prognosis of a breast cancer patient, let alone a case that is further complicated with diabetes. Signature molecular profiling based approach may need to be employed for determining the prognostic outcome of T2DM and breast cancer in a patient. In case of the breast adenocarcinoma samples under study, the expressional status of *TP53*, *IL1β*, *IL8* and *MMP9* indicate an expressional advantage for the patients’ prognosis, while on the other hand the *IL6*, *CTNNB1* and *MYC*’s expression patterns may prove as a disadvantage. The clinically relevant question that arises is on the extent of influence each of these genes have on disease pathogenesis, T2DM-BC crosstalk and hence on the patient prognosis. Their expression profiling in comorbid condition and correlation with clinico-pathological features may provide further insight into their prognostic relevance.

The reanalysis of these hub genes for functional pathway enrichment, highlighted their role in cancer specific pathways, including breast cancer (a significant enrichment, however not amongst the top 10 most significant results), involving *TP53*, *MYC* and *CTNNB1*, in particular, insulin resistance (*IL6 and NOS3*), *HIF1* signaling pathway (*IL6* and *NOS3*), AGE-RAGE signaling (*IL6*, *NOS3*, *IL1B*), and *PI3K-Akt* pathway (*IL6*, *NOS3*, *MYC*, *TP53*), as shown in S1 Table. Breast cancer sub type specific signaling pathways such as those mediated by *estrogen* and *ErBb* are also enlisted. Additionally, microRNAs in cancer pathway is also amongst the significant results. Previously, regulatory microRNA overlap between T2DM and BC has been highlighted [18]. Furthermore, these hub genes may have common transcription factors, highlighting an overlap in their regulation and the potential for a molecular crosstalk. Amongst the enriched transcription factor terms, hypoxia inducible factor 1 alpha (*HIF1A* -enriched for *TP53*, *CTNNB1* and *MYC*), as shown in Fig 7, has previously been reviewed as pivotal for T2DM-BC association [8].

Since, *TP53*, *MYC* and *IL1β* have a similar expression pattern in both diseases, these may serve as common (diagnostic) biomarkers, hence further study on their potential as molecular switches between T2DM and BC is warranted. Furthermore, the under-expression of *TP53* and *IL1β* and overexpression of MYC in comorbid patients may translate into a poorer prognosis and overall survival outcome. However, the potential prognostic relevance of these hub genes also needs to be validated in a wet lab, before any molecular signature gene sets are designed for the early diagnosis, prognostic determination and therapeutic intervention for T2DM induced breast cancer and breast cancer complicated with diabetes.

Considering the functional niche of each of these genes in both the diseases may further highlight their potential reigning capacity on the T2DM-BC crosstalk. *Tumor protein TP53 (p53)* is a master regulating transcription factor, critically implicated in pivoting a ‘see-saw’ between cell survival and programmed cell death pathways [19]. It responds to oncogenic stress in a severity scaled manner, and orchestrates tumor suppressing activities impacting cell growth, DNA repair, metabolism, cell cycle arrest, senescence and cell death [20]. Hypoxia induces *TP53* mediated suppression of glycolysis and promotion of oxidative phosphorylation [21], supporting its expression mediated favorable prognosis in breast cancer patients. Its regulation of metabolic pathways establishes its role in metabolic diseases beyond cancer, including diabetes. In T2DM, a metabolic disorder, expressional *p53* up-regulation is associated with the regulation of beta cell function [22–24], and is consistent with its expression in T2DM adipose tissue. For T2DM pathogenesis, *TP53* may contribute through multiple routes such as its role in the dysregulation of glucose homeostasis and the development of insulin resistance [25]. In breast cancer, *TP53* is a well-established biomarker. It is reported to be mutated in about 20–40% of all cases and is associated with breast cancer risk and prognosis [26]. However, the up-regulation of its wild type in breast adenocarcinoma samples under study do not indicate an overall poorer prognosis in breast cancer patients, as shown by the Kaplan-Meir plot, hence wet lab validation may be required to fully establish the association of its wild type overexpression with BC prognosis.

*v-Myc*, alternatively known as *c-Myc*, is reported to play a direct role in loss of beta cell mass and impaired insulin secretion [27]. This supports the up-regulation of *c-Myc* in T2DM adipose tissue, as found in this study. Previous studies have shown that *c-Myc* expression is induced by hyperglycemic conditions, which leads to decreased insulin gene transcription [28]. Furthermore, *MYC* is reportedly amplified in about 15% of breast cancer [29], characteristic of various breast cancer subtypes, and associated with poorer prognostic outcome [29]. Its exact role in cancer progression may vary depending on breast cancer molecular subtype, but is associated with endocrine therapy resistance. Additionally, *MYC* has been previously identified as a potential hub gene in diabetes associated complications and their underlying signaling and is implicated in beta cell’s loss of functionality [27, 30].

Moreover, the upregulation of pro-inflammatory cytokines such as interleukins *IL1β*, *IL6* and *IL8*, also known as *CXCL8*, in the T2DM affected adipose tissue, is in line with published reports of their elevated levels and role in the development of T2DM, particularly in causing inflammation and insulin resistance [31]. In particular, *IL-1β* is secreted by pancreatic beta cells under high glucose conditions [32, 33], and is associated with inflammation, beta-cell failure, and its death [34–36]. *IL6* plays a major role in the development of insulin resistance, controlling proliferation, differentiation, migration and apoptosis [37]. Previously, it was identified as a hub gene for T2DM [38]. Similarly, *IL8* is associated with increased cell survival and proliferation, production of matrix metalloproteinases, angiogenesis regulation, and inflammation [39, 40], and has also been reported as a hub gene for T2DM [41]. These pro-inflammatory cytokines are also positively associated with breast cancer progression [42–44]. However, the expression pattern of these cytokines in breast adenocarcinoma samples utilized for this analysis may provide conflict and subsequent research may be necessary to determine the breast cancer subtype specific role of these hub genes.

Moreover, *matrix metalloproteinase 9 (MMP9)* is a known regulator of ECM remodeling, and has been identified as a cancer biomarker associated with poor overall survival [45, 46]. Interestingly, increased *MMP9* levels are also reported in T2DM [47, 48]. Next, *beta–catenin (CTNNB1)* is implicated in beta cell differentiation and function [49], and its pathway’s activation is reported in breast cancer subtypes, particularly triple negative breast cancer [50]. Lastly, *endothelial nitric oxide synthase (NOS3)* is involved in T2DM susceptibility [51], is also implicated in breast cancer, promoting angiogenesis, inflammation, invasion and metastasis [52–55].

Of particular interest, is the combination of *TP53*, *MYC* and *IL1β*. The trio may be a module or sub-network on the molecular map underlying T2DM-BC crosstalk. Although the potential role of these hub genes in T2DM induced BC is also supported by literature, further extensive experimental study is required to test this hypothesis and also validate these predictive results over a larger population size. While the small sample size, particularly for T2DM adipose tissue microarray data is a limiting factor for this study, further availability of freely accessible microarray data in the future may allow for further analyses on a larger scale in silico. Subsequent studies may also be carried out to identify potential markers distinctive to each of BC subtypes, on the road to precision medicine.

Since the nature of this study is focused on in silico approach to explore potential candidates for biomarker discovery associated with T2DM-BC signaling crosstalk, validation of the results was also conducted in insilico. RNA-seq. data for patients with both diabetes and breast cancer was retrieved and a DEG analysis was carried out in reference to paired breast cancer samples. This series with BC samples as control was considered not only because of lack of availability of other analyzable comorbid samples, but also as it was important to study the possible molecular changes in mammary tissue during disease development. A total of 60 genes were differentially expressed in comorbid patients (Fig 8). These included 33 up-regulated DEGs, represented by KEGG pathways for bile and pancreatic secretions and riboflavin metabolism (S1A Fig). The remaining 27 DEGs were down-regulated genes, implicated in KEGG pathways for osteoclast differentiation, *IL-17* and *TNF* signaling (S1B Fig).

Venn diagram analysis to find a common differentially expressed denominator for all three diseased states showed *SIK1*, as the overlapping DEG. *Salt inducible kinase 1 (SIK1)* is an AMP-activated protein kinase, involved in metabolic homeostasis, particularly in glucose and lipid metabolism [56]. Interestingly, it has been implicated negatively in tumorigenesis, development of insulin resistance and is a known negative regulator of osteoblast differentiation. In breast cancer, Its low expression is associated with poor prognosis and is shown to exhibit tumor suppressive properties [57], indicating at the plausible poor prognostic effect of its low expression in the comorbid state; it was down-regulated with respect to its expression in BC samples, analyzed in this study. The prognostic outcome of its low expression is also supported by survival analysis curve, shown in Fig 9D.

Subsequently, DEGs overlapping between comorbid state and T2DM, and similarly its overlap with BC diseased state were studied. It is noteworthy, that with the exception of *ErbB* expression, which was up-regulated in both comorbid D+BC and BC series, the expression pattern of other DEGs showed an inverse correlation between comorbid and single disease T2DM/BC series (Table 4), hinting at possible dysregulations underlying the development of comorbidity, and subsequently mapped into diseased state specific molecular signature overlap, Table 5.

**Table 5:** Disease specific molecular expression signature patterns. Peach color indicates up-regulation whereas teal depicts down-regulation. Grey-no differential expression. Darker color intensity significance statistical significance. DEGs: differentially expressed genes; T2DM: type 2 diabetes mellitus, BC: breast adenocarcinoma, D+BC: diabetes and breast cancer comorbid state.

Diseased States Compared	DEGs	Molecular Signature Overlap		
		T2DM	D+BC	BC
D +BC -T2DM- BC	*SIK1*			
D+BC- T2DM	*FOSB*			
	*IER2*			
	*JUN*			
	*ATF3*			
	*ADCY1*			
	*JUND*			
	*CSRNP1*			
D+BC- BC	*ERRB2*			
	*LBP*			
T2DM- BC	*IL6*			
	*CXCL8*			
	*IL1B*			
	*MYC*			

While the hub genes identified in the cross disease analysis were not amongst the 60 DEGs (adjusted *p* <0.05) obtained from the comorbid series DEG analysis, it is worth mentioning that by applying a less stringent cut off criteria for identifying statistically significant DEGs, i.e. for p < 0.05, 4 out of the 8 hub genes *(IL6*, *CXCL8*, *IL1B* and *MYC)* identified were found to be present in the list of 950 DEGs obtained from the DEG analysis and 12 overlapping DEGs obtained from the Venn diagram analysis (S2 Fig). This may in part be accounted by the small sample size and, given that in the absence of other analyzable RNA-seq. or microarray for comorbid patients, the reliance upon the inclusion of series GSE150586, for the validation became necessary, in hope of achieving as much insight into the potential role of hub and other key genes identified as possible. While it may elucidate on one side of the story, it by no means surpass the significance and necessity of further validation and confirmation of the results of this study in wet lab and on larger study sample size.

**Fig 10:**
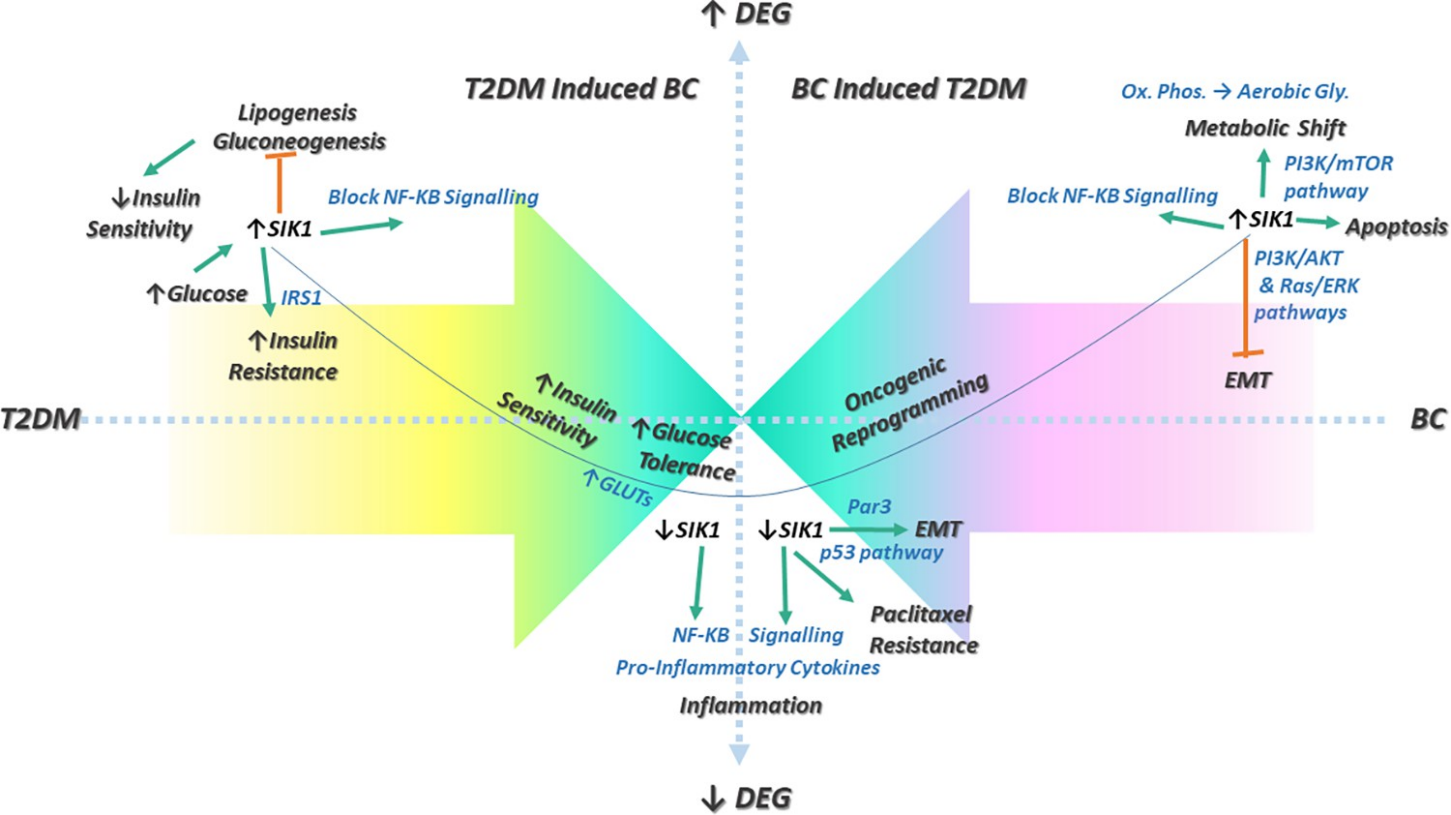
Schematic model for SIK1 mediated T2DM-BC crosstalk leading to comorbidity. The figure highlights the two way relationship between T2DM and BC resulting in T2DM induced BC and BC induced T2DM phenotypes. For either direction, the molecular mechanisms underlying SIK1 expression regulated crosstalk are depicted, predicting possible routes to comorbidity.

Based on the convergence of differentially expressed genes from each diseased state on *SIK1*, as highlighted in Table 4, multifaceted functionality of *SIK1* is predicted and outlined to direct the molecular routes for T2DM induced breast cancer and breast cancer complicated with diabetic onset, modelled in Fig 10.

As shown in the figure, comorbidity can be approached by two distinct directionalities: i. T2DM signaling leading to breast carcinogenesis in mammary tissue and, ii. BC signaling promoting hyperglycemia and insulin resistance characteristic of T2DM. Differential pathways underlying disease pathogenesis may converge at common nodes such as the down-regulation of *SIK1*, leading to immune-metabolic changes contributing to the development of comorbid pathogenic state. In view of the plausible *SIK1* molecular connections derived from published literature, the model could subsequently be implemented and tested in wet lab, to fully elucidate on the role of *SIK1* in establishing the crosstalk between T2DM-BC diseased states.

Taking into account the potentially relevant *IL1B*, *MYC*, *IL6 and CXCL8 hub genes*, *and* additionally, key genes such as *FOSB*, *IER2*, *JUN(D)*, *ATF3*, *ADCY1* and *CSRNP1* common between D+BC and T2DM diseased states, and *ErbB2* and *LBP* common for D+BC and BC states may allow further insights into the molecular interplay between diseased states.

To comprehend their roles in T2DM-BC crosstalk, it is essential to understand the currently known contributions of these additional key genes identified by validation study, to the combinatorial and differential T2DM and BC associated signaling pathways. In continuation with previous discussion on *SIK1*, as depicted in Fig 10, and as supported by pathway enrichment analysis, it is implicated in fasting/hyperglycemia induced silencing of genes responsible for gluconeogenesis [58, 59]. It is also reported as a potentially promising therapeutic target for countering insulin resistance in obesity [60].

Metabolic rewiring is central to not only T2DM but also to breast cancer. *SIK1*, promotes oxidative phosphorylation, hence its down-regulation is reported to promote aerobic glycolysis via *p53/mTOR* signaling [61]. This is consistent with its elevated levels associating favorably with breast cancer OS, as shown in Fig 9D. Moreover, loss of *SIK1* expression facilitates oncogenic transcriptional reprogramming and metastasis [57, 62–64]. It is reported to cause epithelial to mesenchymal transition (EMT) via *Par3*, a polarity protein that regulates tight junctions [65]. Still, further investigation of *SIK1* on T2DM-BC molecular axes may provide greater clarity on the molecular alterations underlying its down-regulation in D+BC phenotype and its clinical outcome, as a consequence.

Moreover, proto-oncogene *FOSB* belongs to the *Fos* family of transcription factors, previously implicated in the hyperglycemia and hypoxia induced *HIF1A*’s modulation of oxidative stress and inflammatory pathways [66]. Further study may help elaborate on its role on the hyperglycemia-hypoxia axis. In breast cancer, *FOSB* is typically down-regulated, and its role in anti-cancer drug treatment and ROS accumulation induced *FOSB* expression mediated cell death has also been studied, however its functionality is still under explored and requires further attention [67]. Interestingly, transcription factor enrichment analysis identified *FOS* as a significant transcription factor implicated in the regulation of hub genes, as shown in Fig 7A, further implying its potential relevance to T2DM-BC crosstalk, worth pursuing for research and validation.

*Immediate early response 2 (IER2)* is also a transcription factor, previously reported to promote metastasis in colon cancer cell lines, and while it is reported to be down-regulated in combination with *FOSB* and *JUN* in breast cancer [68], further to this, its function is not reported for either breast cancer or T2DM. On the other hand, *JUND*, another one of the common DEGs identified between D+BC and T2DM, is a transcription factor belonging to the *JUN* family, known to regulate beta cell functionality and its overexpression is associated with increased lipid accumulation and impaired secretion of insulin in response to glucose [69]. Another study reported its hyperglycemia induced down-regulation and correlation with oxidative stress, elevated *NF-KB* binding and expression of mediators of inflammation [70]. In breast cancer research, its anti-proliferative effect in response to anti-cancer drug has been studied [71].

In addition, *activating transcription factor 3 (ATF3)*, is a crucial for glucolipid metabolism regulation, maintaining metabolic and immune homeostasis and leading to a protective niche against metabolic disorders and oncogenesis [72]. However a recent study on ATF3 as an endoplasmic reticulum stress/injury marker, reported it to induce neuro-inflammation in diabetic neuropathy [73], and another studied its role in promoting the expression of tumor metastatic genes such as *fibronectin* and *MMP13*, amongst others [74]. It is also shown to regulate cell proliferation and induce resistance against radiation therapy, implicating the *PI3K-AKT* pathway. Hence its multi-faceted role may further be explored in context of T2DM-BC crosstalk.

Moreover, *cysteine rich nuclear protein 1 (CSRNP1)* another key gene identified, has been associated with the reduced glomerular filtrate rate, affecting kidney function in diabetes, in a recently published GWAS study [75]. However its implication on T2DM-BC crosstalk is not known and requires study. *Adenylate cyclase 1 (ADCY1)*, a reported prognostic marker for pancreatic and lung cancers [76], may also be pursued for its role in breast cancer, which is currently underexplored. Previously, it was shown to promote *ERK* mediated cell death in response to doxorubicin, in breast cancer cells [77], hence potentiating its relevance as a prognostic marker for breast cancer. Although its expression is found to be up-regulated in this analysis, its expression showed no correlation with T2DM in a previously published study [78]. Further investigation into its role in T2DM and comorbid state may elucidate on its potential as a biomarker for T2DM induced breast cancer, along with other key genes discussed.

Furthermore, the key genes identified for breast cancer with T2DM onset include *ErbB2* and *LBP*. *ErbB2*, more widely known as *human epidermal growth factor receptor 2* (*HER2*) is a tyrosine kinase, reportedly overexpressed in 25–30% of breast cancer cases in humans, and strongly associated with malignancy and invasiveness [79, 80]. Its role in breast cancer is extensively studied and established it as a diagnostic biomarker for *HER2* expressing breast cancer subtypes, prognostic marker reflecting poor prognosis and recurrence and as a therapeutic target for immunotherapy against breast cancer. Hence, its up-regulation in D+BC and BC series indicate at *HER2* positive expression subtype/s. Its role in diabetes has also been explored. A population based cohort study associated *ErbB2* expression with increased incidence of diabetes, based on its significant correlation with insulin, glucose and HbA1c levels [81]. This is in line with the results of this study identifying it as a potential biomarker for BC induced T2DM phenotype. Similarly, *Lipopolysaccharide binding protein (LBP)* was reported as a prognostic biomarker for breast cancer, insightful of the effect of radiation therapy on cardiac dysfunction [82]. Interestingly, it is also associated with prognosis in T2DM patients, particularly with arterial stiffness [83]. Hence, common differentially expressed genes between two or more diseased state may indicate at their role within the T2DM-BC signaling converging network, however, whether these key genes are centrally situated within the T2DM-BC crosstalk and what is the outcome of their differential expression remains to be elucidated, rendering subsequent wet lab studies crucial.

For this study, it is important to note however, that the status of DEGs in D+BC state is subject to its expression in its BC counterpart samples, hence a down-regulation in *SIK1* expression, for instance is only relative to its expression in breast cancer. The comparison of D+BC samples with healthy controls and also with T2DM patients is still needed to provide further insights and to reflect on a completed puzzle, however in the absence of such analyzable datasets publically available, this study relies on interpreting the effect of T2DM crosstalk with BC on mammary tissue from the perspective of altered breast cancer specific expression when complicated with T2DM. Other factors such as the order of occurrence of the diseases in comorbidity and the subtype of breast cancer and diabetes may also affect the analysis and provide further clarity, when taken into account, for further study.

## Conclusion

Type 2 diabetes mellitus (T2DM) and breast cancer (BC)’s two-way relationship is multi-faceted, affecting patient diagnosis, prognosis and treatment. The underlying molecular mechanisms may involve an intricately knitted crosstalk of several signaling pathways implicated in the pathogenesis of both diseases. Epidemiological data till date establishes a moderate yet significant association between the two complex diseases, however experimental data is necessary to validate the association and its underlying molecular mechanisms. This study, reports *SIK1* as a potentially crucial gene to T2DM-BC crosstalk, and outlines plausible mechanisms underlying its potential role in T2DM-BC association at molecular level. It additionally identifies 8 hub genes common to both T2DM and BC, and of which *TP53*, *MYC* and *IL1β* were found to exhibit similar expression patterns in both diseases, highlighting their potential as common biomarkers for T2DM- BC crosstalk.

However, further analysis of larger sample size data collected from patients with both diabetes and breast cancer to identify a conserved signature gene set common to all cases, followed by wet lab validation is necessary to provide further insights into the interactive network of molecular players crucially implicated in diabetes-breast cancer crosstalk.

## Supporting information

S1 Fig Pathway enrichment analysis. The most significant KEGG pathway results are presented from top to bottom for common DEGs between all three series (p <0.05) **A**- Up-regulated DEGs, **B**- Down-regulated DEGs.
(TIF)

S2 Fig Venn diagram analysis for diabetes and breast cancer (D+BC) comorbid samples with T2DM -adipose tissue and breast adenocarcinoma (BC) samples. The yellow circle represents DEGs obtained from DEG analysis (p < 0.05) on T2DM samples, pink circle represents DEGs from BC samples, and sea green circle depict DEGs for D+BC samples, similarly. **A**- The intersection represents DEGs common to D+BC, T2DM and BC series. **B**- The intersection represents DEGs common to D+BC and T2DM diseased conditions. **C**- The intersection represents DEGs common D+BC and BC diseased states.
(TIF)

S1 Table KEGG analysis of hub genes. The table lists the top ten significant terms, along with other terms potentially relevant to T2DM-BC Crosstalk.
(DOCX)

## List of abbreviations

*LGALS8*: galectin 8
*S100A12*: S100 calcium binding protein A12
*KDM5D*: lysine demethylase 5D
*MIOX*: myo-inositol oxygenase
*ISLR*: immunoglobulin superfamily containing leucine rich repeat
*VNN2*: vanin 2
*CAMK2B*: calcium/calmodulin dependent protein kinase II beta
*GP9*: glycoprotein IX platelet
*NEUROD2*: neuronal differentiation 2
*FOS*: Fos proto-oncogene, AP-1 transcription factor subunit
*SPATS2L*: spermatogenesis associated serine rich 2 like
*PLXNA4*: plexin A4
*SPDYE3*: speedy/RINGO cell cycle regulator family member E3
*SP1*: Sp1 transcription factor
*DOK5*: docking protein 5
*TAX1BP3*: Tax1 binding protein 3
*ARHGAP26*: Rho GTPase activating protein 26
*ZSCAN29*: zinc finger and SCAN domain containing 29
*ADAMTS10*: ADAM metallopeptidase with thrombospondin type 1 motif 10
*TERT*: telomerase reverse transcriptase
*FRG2B*: FSHD region gene 2 family member B
*CD207*: CD207 molecule
*AMPD2*: adenosine monophosphate deaminase 2
*ZNF8*: zinc finger protein 85
*SLC24A5*: solute carrier family 24 member 5
*SLC5A12*: solute carrier family 5 member 12
*FBXO33*: F-box protein 33
*KRTAP23*: *1* keratin associated protein 23–1
*ABCB4*: ATP binding cassette subfamily B member 4
*A1CF*: APOBEC1 complementation factor
*SHCBP1*: SHC binding and spindle associated 1
*SAA4*: serum amyloid A4, constitutive
*LINC00152*: *long intergenic non-protein coding RNA 152*
*MIGA1*: mitoguardin 1
*EYA4*: EYA transcriptional coactivator and phosphatase 4
*CHADL*: chondroadherin like
*CDK15*: cyclin dependent kinase 15
*EXOC7*: exocyst complex component 7
*CCL23*: C-C motif chemokine ligand 23
*PYROXD1*: pyridine nucleotide-disulphide oxidoreductase domain 1
*SOD3*: superoxide dismutase 3, extracellular
*POMZP3*: POM121 and ZP3 fusion
*TAPBP*: TAP binding protein (tapasin)
*MCL1*: BCL2 family apoptosis regulator
*CRTC1*: CREB regulated transcription coactivator 1
*MMP9*: matrix metallopeptidase 9
*CXCL8*: C-X-C motif chemokine ligand 8
*MAP2K3*: mitogen-activated protein kinase kinase 3
*SERPINB2*: serpin family B member 2
*MYC*: v-myc avian myelocytomatosis viral oncogene homolog; alternatively c-Myc
*MSR1*: macrophage scavenger receptor 1
*S100A8*: S100 calcium binding protein A8
*CEL*: carboxyl ester lipase
*TPM4*: tropomyosin 4
*ELN*: elastin
*PTMS*: parathymosin
*SLC11A1*: solute carrier family 11 member 1
*RBMS1*: RNA binding motif single stranded interacting protein 1
*TPM3*: tropomyosin 3
*ZC3HAV1*: zinc finger CCCH-type containing, antiviral 1
*TNC*: tenascin C
*WDR1*: WD repeat domain 1
*FGFR1*: fibroblast growth factor receptor 1
*MAPK8IP3*: mitogen-activated protein kinase 8 interacting protein 3
*RP2*: *retinitis pigmentosa 2 (X-linked recessive)*
*CALD1*: caldesmon 1
*AGRN*: agrin
*GEM*: GTP binding protein overexpressed in skeletal muscle
*ARID4B*: AT-rich interaction domain 4B
*MRGPRF*: MAS related GPR family member F
*TYMP*: thymidine phosphorylase
*SIK1*: salt inducible kinase 1
*ACTRT1*: actin related protein T1
*HIPK2*: homeodomain interacting protein kinase 2
*SCAMP1*: secretory carrier membrane protein 1
*FSTL3*: follistatin like 3
*ITGAX*: integrin subunit alpha X
*CCDC86*: coiled-coil domain containing 86
*ADAMTS1*: ADAM metallopeptidase with thrombospondin type 1 motif 1
*SP2*: Sp2 transcription factor
*SYN2*: synapsin II
*PRMT2*: protein arginine methyltransferase 2
*SNCA*: synuclein alpha
*PMP22*: peripheral myelin protein 22
*RFX1*: regulatory factor X1
*CDKN1A*: cyclin dependent kinase inhibitor 1A
*NOS3*: nitric oxide synthase 3
*NUP98*: nucleoporin 98
*EPOR*: erythropoietin receptor
*LDOC1L*: leucine zipper down-regulated in cancer 1 like
*DYNLT3*: dynein light chain Tctex-type 3
*TP53*: tumor protein p53
*GGCX*: gamma-glutamyl carboxylase
*WTAP*: Wilms tumor 1 associated protein
*SF3A2*: splicing factor 3a subunit 2
*GRASP*: general receptor for phosphoinositides 1 associated scaffold protein
*PTPRE*: protein tyrosine phosphatase, receptor type E
*CYSLTR1*: cysteinyl leukotriene receptor 1
*EPN1*: epsin 1
*BCORL1*: BCL6 corepressor-like 1
*PLPP1*: phospholipid phosphatase 1
*WDR4*: WD repeat domain 4
*TUBA1C*: tubulin alpha 1c
*HSPA1A*: heat shock protein family A (Hsp70) member 1A
*RBPJ*: recombination signal binding protein for immunoglobulin kappa J region
*FAM222B*: family with sequence similarity 222 member B
*CBX4*: chromobox 4
*FKBP1A*: FK506 binding protein 1A
*FGR*: FGR proto-oncogene, Src family tyrosine kinase
*CTNNB1*: catenin beta 1
*IL1B*: interleukin 1 beta
*WRNIP1*: Werner helicase interacting protein 1
*MYL9*: myosin light chain 9
*TSPAN13*: tetraspanin 13
*JUNB*: JunB proto-oncogene, AP-1 transcription factor subunit
*STX11*: syntaxin 11
*BCL3*: B-cell CLL/lymphoma 3
*SLC25A20*: solute carrier family 25 member 20
*IL6*: interleukin 6
*GTPBP8*: GTP binding protein 8 (putative)
*EXOC4*: exocyst complex component 4
*CDC14A*: cell division cycle 14A
*SMAD5*: SMAD family member 5
*KRTAP3*: *2* keratin associated protein 3–2
*PER3*: period circadian clock 3
*MCOLN3*: mucolipin 3
*RPL21*: ribosomal protein L21
*CHURC1*: churchill domain containing 1
*EGFR*: epidermal growth factor receptor
*TFAP2A*: transcription factor AP-2 alpha
*SMAD3*: an acronym from the fusion of Caenorhabditis elegans Sma genes and mothers against decapentaplegic transcription factor 3
*KAT2A*: protein lysine acyltransferase
*CEBPA*: CCAAT enhancer-binding protein alpha
*HIF1A*: hypoxia inducible factor 1-alpha
*TFAP2C*: transcription factor AP-2 gamma
*EP300*: histone acetyltransferase p300
