# Supplementary figures and images for: Integrated bioinformatics analyses identifying potential biomarkers for type 2 diabetes mellitus and breast cancer: In SIK1-ness and health
Source: PLoS One. 2023 Aug 9;18(8):e0289839. doi: 10.1371/journal.pone.0289839 (PMC10411810; doi:10.1371/journal.pone.0289839)

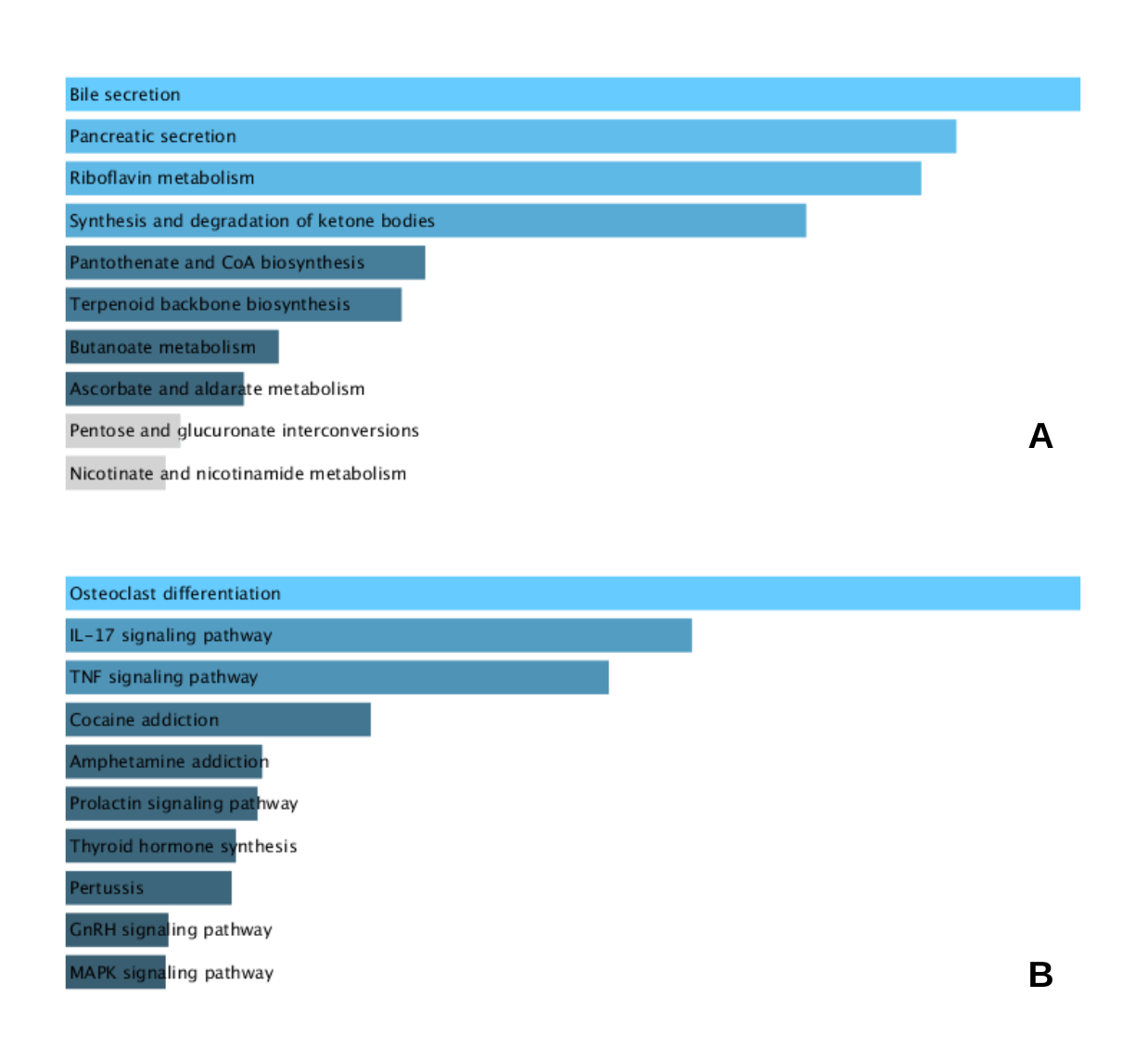

Supplement: S1 Fig — The most significant KEGG pathway results are presented from top to bottom for common DEGs between all three series (p <0.05) A- Up-regulated DEGs, B- Down-regulated DEGs. (TIF) [file pone.0289839.s001.tif]

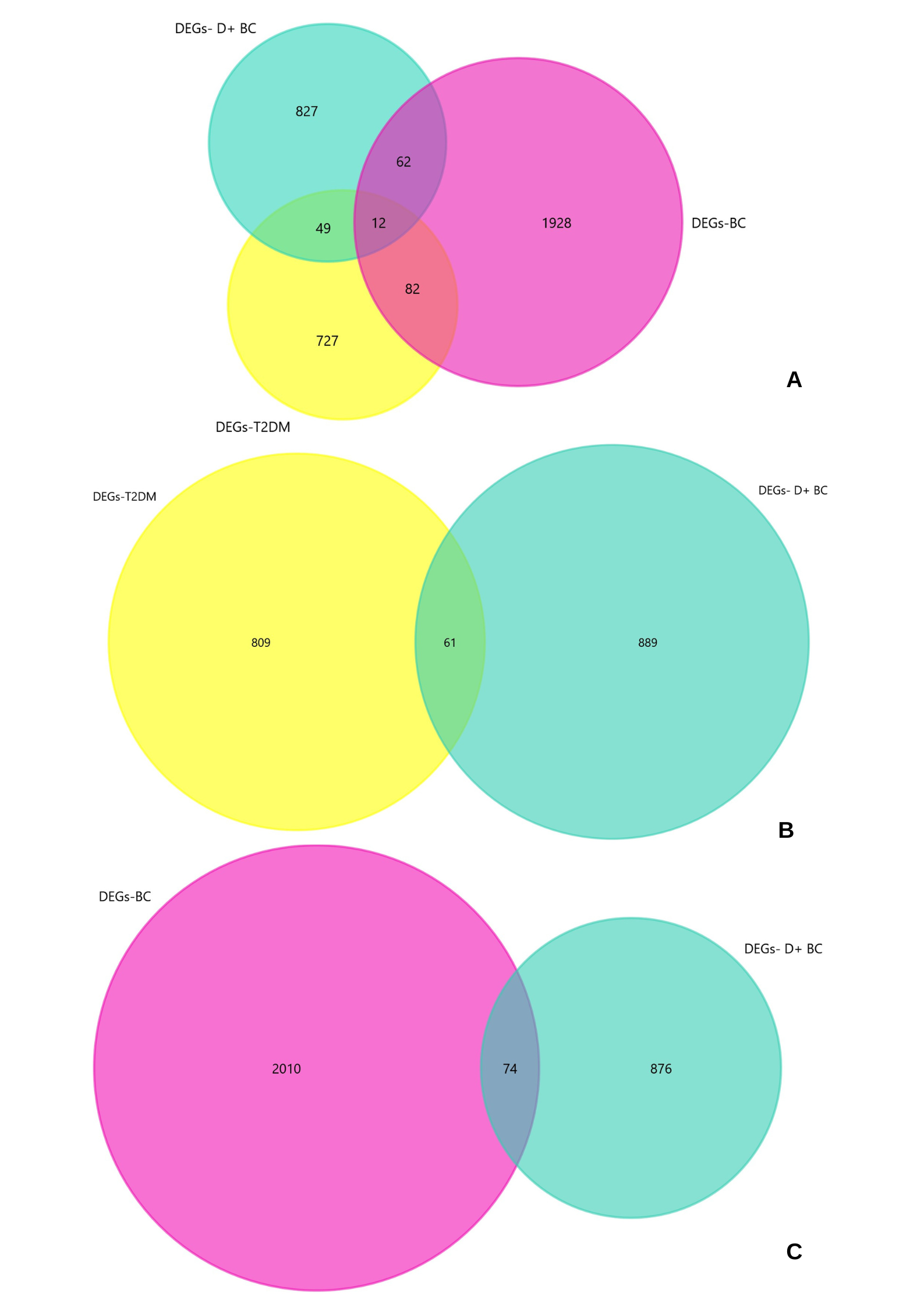

Supplement: S2 Fig — The yellow circle represents DEGs obtained from DEG analysis (p < 0.05) on T2DM samples, pink circle represents DEGs from BC samples, and sea green circle depict DEGs for D+BC samples, similarly. A- The intersection represents DEGs common to D+BC, T2DM and BC series. B- The intersection represents DEGs common to D+BC and T2DM diseased conditions. C- The intersection represents DEGs common D+BC and BC diseased states. (TIF) [file pone.0289839.s002.tif]
