# Supplementary material for: Integrated bioinformatics analyses identifying potential biomarkers for type 2 diabetes mellitus and breast cancer: In SIK1-ness and health
Source: PLoS One. 2023 Aug 9;18(8):e0289839. doi: 10.1371/journal.pone.0289839 (PMC10411810; doi:10.1371/journal.pone.0289839)
Supplement: S1 Table — The table lists the top ten significant terms, along with other terms potentially relevant to T2DM-BC Crosstalk. (DOCX) [file pone.0289839.s003.docx]

| **Category** | **KEGG Pathway Term** | **Adjusted *p* Value** | **Genes** |
| --- | --- | --- | --- |
| Top ten significant terms | Fluid shear stress and atherosclerosis | 9.63E-08 | *NOS3; IL1B; CTNNB1; TP53; MMP9* |
|  | Lipid and atherosclerosis | 3.63E-07 | *IL6; NOS3; IL1B; TP53; MMP9* |
|  | Human cytomegalovirus infection | 3.63E-07 | *IL6; IL1B; MYC; CTNNB1; TP53* |
|  | Hepatitis B | 7.52E-06 | *IL6; MYC; TP53; MMP9* |
|  | Thyroid cancer | 7.52E-06 | *MYC; CTNNB1; TP53* |
|  | Bladder cancer | 8.28E-06 | *MYC; TP53; MMP9* |
|  | Transcriptional misregulation in cancer | 8.28E-06 | *IL6; MYC; TP53; MMP9* |
|  | Kaposi sarcoma-associated herpesvirus infection | 8.28E-06 | *IL6; MYC; CTNNB1; TP53* |
|  | Pathways in cancer | 8.43E-06 | *IL6; MYC; CTNNB1; TP53; MMP9* |
|  | Proteoglycans in cancer | 8.43E-06 | *MYC; CTNNB1; TP53; MMP9* |
| Other relevant significant terms | *IL-17* signaling pathway | 4.55E-05 | *IL6; IL1B; MMP9* |
|  | *PI3K-Akt* signaling pathway | 4.55E-05 | *IL6; NOS3; MYC; TP53* |
|  | AGE-RAGE signaling pathway in diabetic complications | 4.55E-05 | *IL6; NOS3; IL1B* |
|  | *TNF* signaling pathway | 6.04E-05 | *IL6;IL1B;MMP9* |
|  | Breast cancer | 1.16E-04 | *MYC; CTNNB1; TP53* |
|  | Cellular senescence | 1.25E-04 | *IL6; MYC; TP53* |
|  | *Wnt* signaling pathway | 1.41E-04 | *MYC; CTNNB1; TP53* |
|  | *MAPK* signaling pathway | 6.16E-04 | *IL1B; MYC; TP53* |
|  | MicroRNAs in cancer | 6.85E-04 | *MYC; TP53; MMP9* |
|  | Insulin resistance | 0.001765 | *IL6; NOS3* |
|  | *HIF-1* signaling pathway | 0.001765 | *IL6; NOS3* |
|  | *Estrogen* signaling pathway | 0.002496 | *NOS3; MMP9* |
|  | *JAK-STAT* signaling pathway | 0.003296 | *IL6; MYC* |
|  | Diabetic cardiomyopathy | 0.004704 | *NOS3; MMP9* |
|  | *VEGF* signaling pathway | 0.034303 | *NOS3* |
|  | *p53* signaling pathway | 0.039173 | *TP53* |
|  | *ErbB* signaling pathway | 0.042709 | *MYC* |

***S1 Table***  **KEGG Analysis of Hub Genes***-* The table lists the top ten significant terms, along with other terms potentially relevant to T2DM-BC Crosstalk.
